# Tuning the upconversion photoluminescence lifetimes of NaYF_4_:Yb^3+^, Er^3+^ through lanthanide Gd^3+^ doping

**DOI:** 10.1038/s41598-018-30983-9

**Published:** 2018-08-23

**Authors:** Heng Qin, Danyang Wu, Juna Sathian, Xiangyu Xie, Mary Ryan, Fang Xie

**Affiliations:** Department of Materials and London Centre for Nanotechnology, Imperial College London, Exhibition Road, London, SW7 2AZ UK

## Abstract

The multiplexing capacity of conventional fluorescence materials are significantly limited by spectral overlap and background interference, mainly due to their short-lived fluorescence lifetimes. Here, we adopt a novel Gd^3+^ doping strategy in NaYF_4_ host materials, realized tuning of upconversion photoluminescence (UCPL) lifetimes at selective emissions. Time-correlated single-photon counting (TCSPC), was applied to measure the photoluminescence lifetimes accurately. We demonstrated the large dynamic range of lifetimes of upconversion nanoparticles with good upconversion quantum yields, mainly owing to the dominance of high efficient energy transfer upconversion mechanism. The exceptional tunable properties of upconversion materials allow great potential for them to be utilized in biotechnology and life sciences.

## Introduction

Upconversion nanoparticles, capable of displaying high-energy luminescent emission through absorption of two or more low-energy photons, have attracted tremendous research attention in past decade due to their remarkable and unique optical properties including sharp emission, low background signal, long decay time *et al*.^1,2^. UCNPs have been widely exploited in many emerging applications such as biological imaging, molecular detection, drug delivery and optoelectronics^3,4^. In particular, with versatile surface modification strategies, it has been demonstrated biocompatibility of UCNPs, significantly boosting their prospect for biomedical applications^1^. Optical multiplexing gains significant attention in biotechnology and life science due to the capability of identifying and quantifying multiple biomolecular species^5^. To realize efficient multiplexing, one of the great challenges is the exploration of optical markers possessing a matrix of optical codes, which could be identified with minimal time, high sensitivity and accuracy. However, conventional fluorescence materials, including quantum dots and organic dyes, only exhibit lifetimes in the order of nanoseconds^6^ and are simply too short for the temporal identification of fluorescence interference from scattered excitation photons. In contrast to these short-lived fluorescence counterparts, lanthanide ions doped upconversion nanoparticles (UCNPs) demonstrate distinct photoluminescence lifetimes from microseconds to even milliseconds^7,8^. Upconversion photoluminescence nanoparticles, not only can realize frequency conversion via converting two (or more) low-energy photons into one high-energy photon, but also exhibit unique optical proper- ties such as high resistance to optical blinking, sharp and multiple luminescence peaks, as well as high photostability^9^. All these advantages make UCNPs a great candidate for multiplexing, allowing the creation of extra coding dimensions. Current research has already suggested that Ln-doped fluorides (i.e., NaYF_4_:Yb^3+^ 20%, Er^3+^ 2%) are the most promising and efficient nanomaterial to achieve desirable UCPL^10–12^. However, poor upconversion efficiencies of UCNPs remains to be one of the most critical limitation for a range of applications^13,14^.

Further studies of the enhancement of upconversion quantum yield (UCQY) of UCNPs are needed to fulfil their full potential for practical applications. Recent advances demonstrated the availability of systematically tuning various properties of UCNPs, including phase, morphology, and quantum yield through designing lanthanide doping strategy and novel nanostructure, such as core-shell structure^15^, nanoarrays structure^16^, etc. Liu realized simultaneous phase and size control of UCNPs with strong UCPL by additional doping of Gd^3+^ ions^17^. Despite these progresses have been achieved, it remains a challenge to precisely tune the UCPL lifetimes of NaYF_4_ nanocrystals, with high efficiency UCPL to overcome the limitation in emission selectivity for the multiplexed applications.

**Figure 1:**
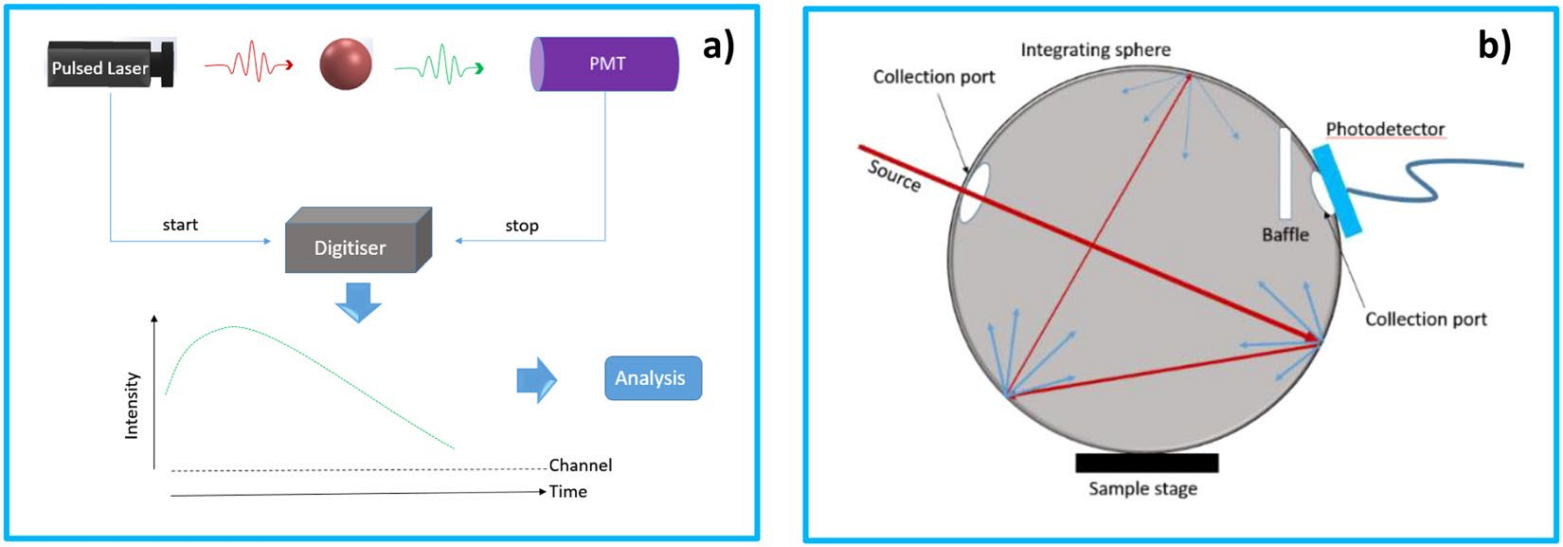
(**a**) Schematic diagram for TCSPC setup. (**b**) Schematic diagram of the integrating sphere setup to measure the quantum yield values.

In this work, we developed a facile Gd^3+^ doping strategy in a hydrothermal process to synthesize a set of upconversion nanomaterials with distinct luminescent properties. The dominant role of energy transfer upconversion (ETU) process in Sensitizer–Activator-coupled UCNPs system was identified by accurate lifetime measurement (TCSPC), as shown in Fig. 1a. Accordingly, we observed that the lanthanide Gd^3+^ ion doping approach leads to a clear prolonged lifetimes of photoluminescence, and the lifetimes of both green emission (at 540 nm) and red emission (at 656 nm) exhibit as a function of the internal upconversion quantum yield (iUCQY), shown in Fig. 1b. In addition, X-ray power diffraction (XRD), field emission scanning electron microscopy (FE-SEM), and were used to characterize the UCNPs. Our results indicate that tuneable UCPL lifetimes have remarkable potential for optical multiplexing applications.

## Methods

Time-correlated single-photon counting (TCSPC), which is considered as the most sensitive digital technique for determining photoluminescence lifetime to date, was used to obtain a dynamic picture of the upconversion photoluminescence of the as-prepared samples^18^. With well-defined Poisson statistics, TCSPC is a method based on the detection of the arrival time of individual photon after optical excitation of a sample. Importantly, this mechanism allows only one photon can be counted at any one time and the measured lifetimes are not affected by changes in source intensity. It is worth to mention that the lifetimes of UCNPs in general cases are independent of excitation power density in the low power regime (<100 *Wcm*^−2^)^19^. We precisely modulated the pulse duration of the 980 nm pulsed laser to statistically characterize the time dependent photoluminescence emission profiles of the as-synthesized UCNPs by repeating the excitation-emission process to 10000 counts.

## Results

**Figure 2:**
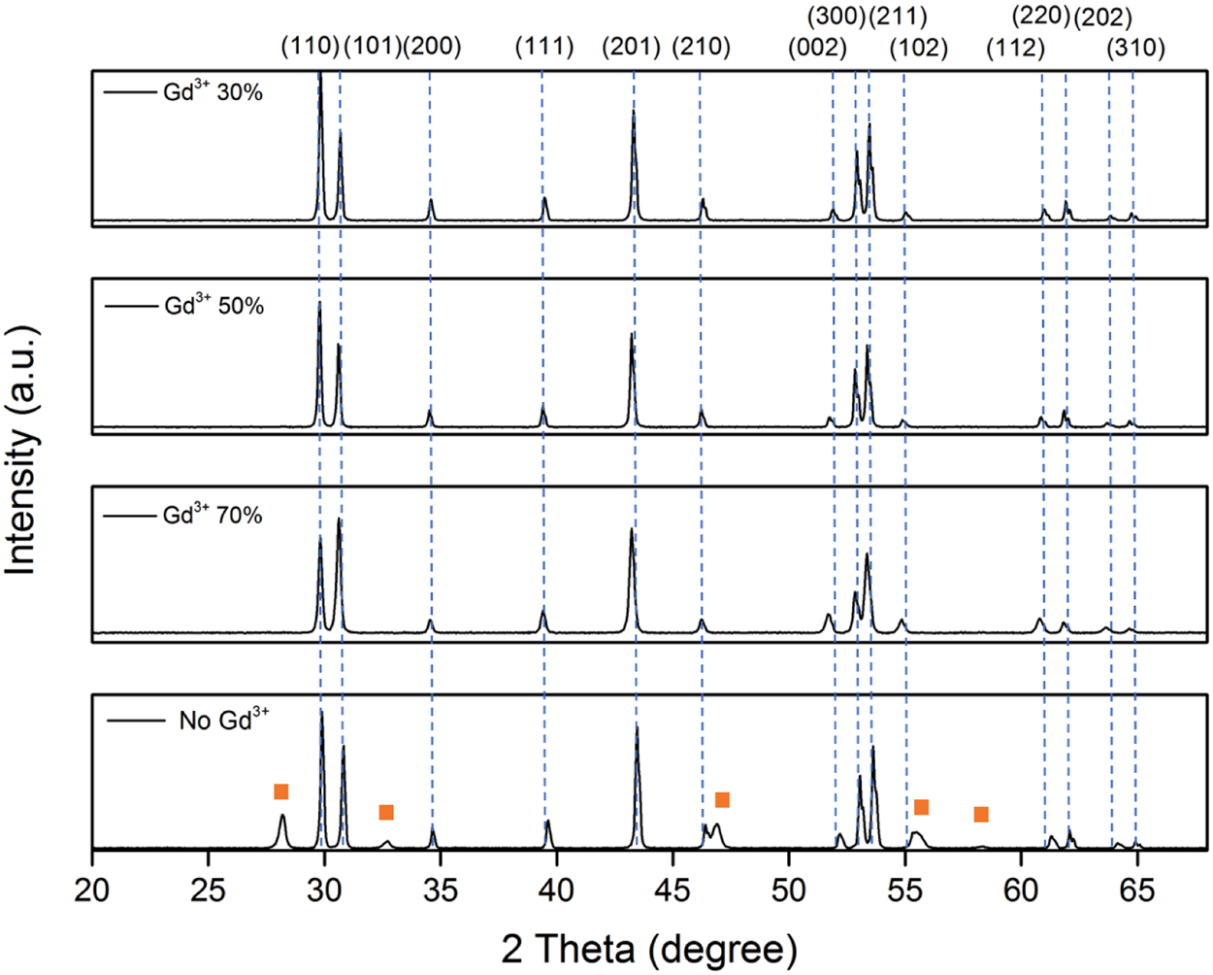
XRD patterns of NaYF_4_ doped with 0 mol%–70 mol% ions and synthesized by a hydrothermal method heated under 200 for 20 hours. The orange squares represent cubic phase of NaYF_4_ while the remaining diffraction peaks represent hexagonal phase.

All samples were first examined by X-ray powder diffraction. Figure 2 shows the XRD patterns of NaYF_4_:Yb^3+^, Er^3+^ materials doped with 0–70% Gd^3+^ ions synthesized at 200 *C* for 20 hours. The diffraction spectra show evidence of the co-existence of cubic phase (JCPDS File No. 772042) and hexagonal phase NaYF_4_ (JCPDS File No. 16-0334) without Gd^3+^ doping prepared in this synthesis condition^17^. Notably, the XRD patterns demonstrate the formation of hexagonal phase NaYF_4_:Yb^3+^, Er^3+^ when doping additional 30% Gd^3+^ according to standard hexagonal XRD spectra of NaYF_4_. This suggests extra dopant Gd^3+^ ions can effectively induced an cubic to hexagonal phase transition under this synthesize condition. No extra diffraction peaks appeared with further increase of the Gd^3+^ concentration from 30 mol% to 70 mol%, which implies the formation of a homogeneous Y-Gd solid solution. Since the Y^3+^ ions were substituted by larger Gd^3+^ ions in the host lattice, the diffraction peaks shifted to lower diffraction angles with the increasing of Gd^3+^ ions concentration and the expansion of unit-cell volume.

**Figure 3:**
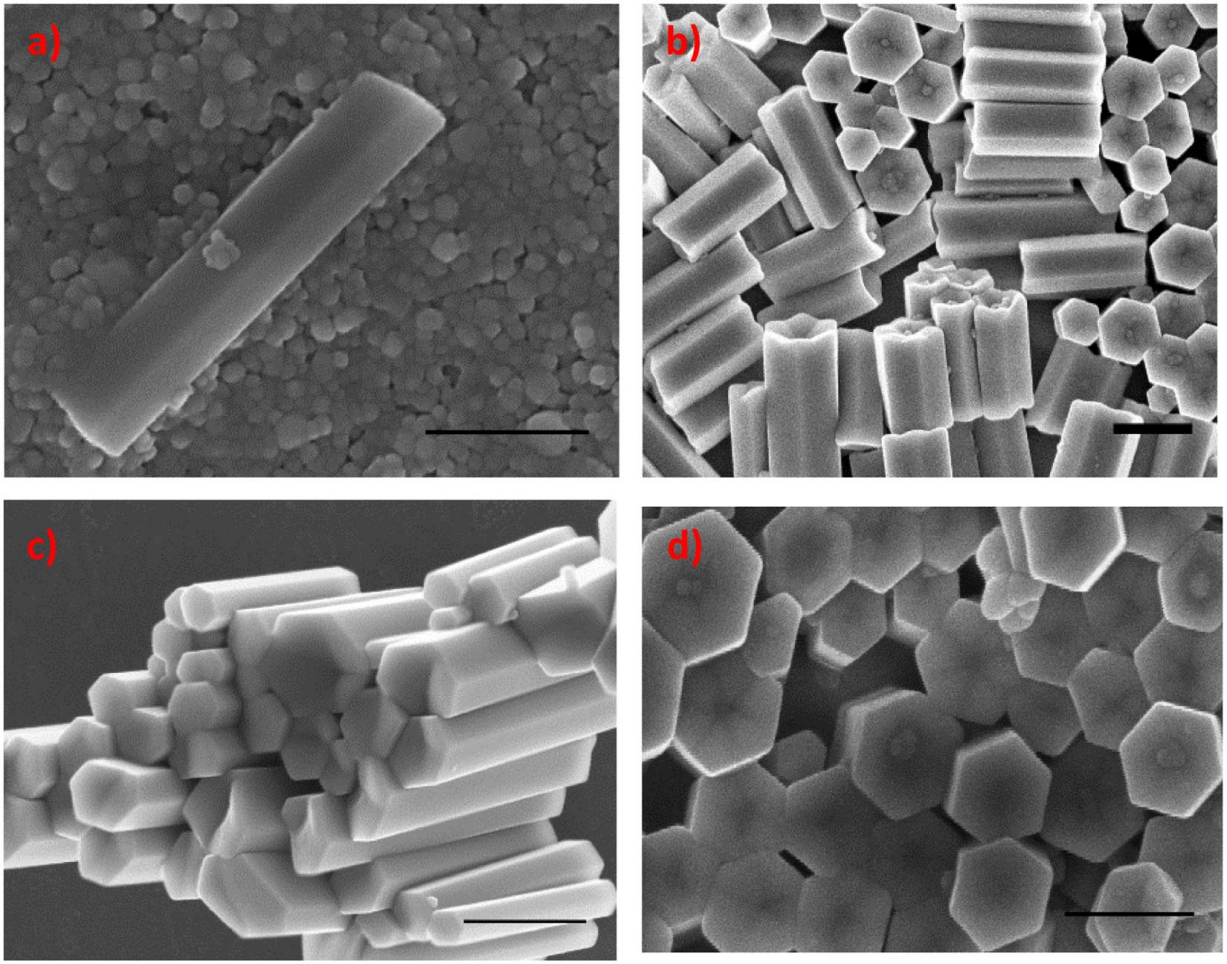
(**a**–**d**) SEM characterization of NaYF_4_:Yb^3+^, Er^3+^ (20, 2 mol%) nanoparticles doped with various concentrations of Gd^3+^ ions. (**a**) shows the SEM image of the NaYF_4_:Yb^3+^, Er^3+^ (20, 2 mol%) UC nanoparticles. (**b**) shows the SEM image of the NaYF_4_:Yb^3+^, Er^3+^ (20, 2 mol%) @Gd^3+^ doping (30 mol%) UC nanoparticles. (**c**) shows the SEM image of the NaYF_4_:Yb^3+^, Er^3+^ (20, 2 mol%) @Gd^3+^ doping (50 mol%). (**d**) shows the SEM image of the NaYF_4_:Yb^3+^, Er^3+^ (20, 2 mol%) @Gd^3+^ doping (70 mol%) UC nanoparticles. The scale bars in figure (**a**–**d**) are 500 nm.

To study the crystallite size and phase of NaYF_4_:Yb^3+^, Er^3+^ doped with different concentrations of Gd^3+^ ions, all the samples were synthesized under a same heating condition and characterized by scanning electron microscopy (SEM), respectively. A brief summary of the synthetic parameters of NaYF_4_:Yb^3+^, Er^3+^, Gd^3+^ materials are listed in Table S1. The influences of Gd^3+^ doping on crystallite size distribution and crystal phase of NaYF_4_:Yb^3+^, Er^3+^ nanoparticles are listed in Table S2. Figure 3a confirmed the co-existence of hexagonal phase and cubic phase NaYF_4_. Clearly, there were significant differences on morphology as a function of the dopant con- centration of Gd^3+^. Figure 3(b) demonstrated that pure hexagonal phase of NaYF_4_ was formed when the Gd^3+^ ion concentration reached 30 mol%, and this as-prepared sample was of good crystallite size uniformity. In addition, the length of the nanorods decreased gradually when the Gd^3+^ doping concentration increased from 30 mol% to 70 mol%. The transition from cubic phase to hexagonal phase could be well controlled by modulating the doping concentration of Gd^3+^.

In view of geometry of UCNPs, 30 mol% Gd^3+^ doped sample shows larger physical dimen- sion, smaller surface defect, high crystallinity and a smaller ratio of surface area to volume. On the contrary, both 50 mol% and 70 mol% Gd^3+^ doping samples have a relatively smaller size, less homogenous morphology, and lower crystallinity. High Gd^3+^ concentrations are associated with an increase of unwanted surface impurities, ligands and lattice defects, which could change the ori- gin phonon energy of the host matrix. Moreover, the size ranges become broader when the Gd^3+^ doping content is raised from 30 mol% to 70 mol%, indicates the tendency of less homogeneity with a higher concentration of lanthanide doping. Notably, when the Gd^3+^ ions are doped into the NaYF_4_:Yb^3+^, Er^3+^ nanoparticles, hexagonal tubes have a protruding centre and distortional tubular structure with the end face convex in the centre and concave between the centre and the edge. When the Gd^3+^ doping concentration is 30 mol%, and F^*−*^/Ln^3+^ molar ratio is calculated as 8:1, the obtained UCNPs are the most uniform ones and have the smoothest morphologies among the three lanthanide doping samples. The evolution of morphology can be partly attributed to the surface modification effect of dopant Gd^3+^ ions on crystal growth^20^.

**Figure 4:**
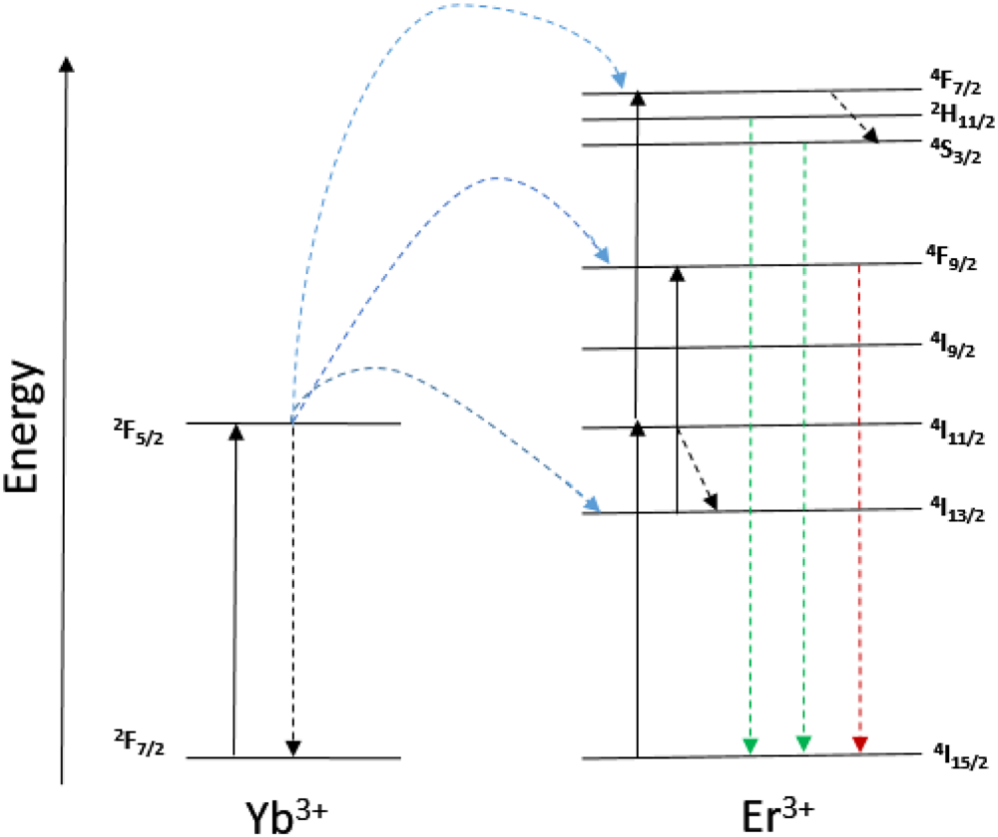
Energy level diagram and upconversion mechanism for the Yb^3+^ and Er^3+^ co- doped UCNPs system upon 980 nm laser excitation. Three major emission peaks centered at 520 nm, 540 nm, and 656 nm are observed in the range of UV to visible light spectrum, where two green emissions at 520 nm and540 nm are assigned to the ^2^H_11*/*2_^4^I_15*/*2_ and ^4^S_3*/*2_^4^I_15*/*2_ transition, respectively; and the red emission at 656 nm is assigned to the ^4^F_9*/*2_^4^I_15*/*2_ transition. The UCPL of Er^3+^ and Yb^3+^ ions co-doped nanoparticles excited by a 980 nm laser emit yellowish green light, which is a combination of green and red colour emissions.

In a typical Sensitizer–Activator-coupled UCNPs system (shown in Fig. 4), ground state absorption/excited state absorption (GSA/ESA), energy transfer upconversion (ETU) and cooperative sensitization (CS) are three main mechanisms for upconversion^21^. ETU, which comprises multiple competing transitions between multiple energy levels in the Yb^3+^ - Er^3+^ couples system, is considered to be the most efficient mechanisms in upconversion process^22,23^. NaYF_4_:Er^3+^,Yb^3+^ exhibits a combination of ESA and ETU process. Er^3+^ ion is excited from the ground-state ^4^I_15/2_ to the excited-state ^4^I_11/2_ by one of the two following processes: ground-state absorption by absorbing one 980 nm laser photon (GSA), or energy transfer (ET) from the excited Yb^3+^ ions

1 $${}^{{\rm{4}}}{\rm{I}}_{{\rm{15}}/{\rm{2}}}({\rm{Er}})+{}^{{\rm{2}}}{\rm{F}}_{{\rm{5}}/{\rm{2}}}(\mathrm{Yb})\to {}^{{\rm{4}}}{\rm{I}}_{{\rm{11}}/{\rm{2}}}(\mathrm{Er})+{}^{{\rm{2}}}{\rm{F}}_{{\rm{7}}/{\rm{2}}}({\rm{Yb}})$$

Then, the ions in the ^4^I_11/2_ state can be immediately excited to the ^4^F_7/2_ level of Er^3+^ ions by absorbing another Yb^3+^ ion. The Er^3+^ ions could decay non-radiatively to the luminescent states ^2^H_11/2_^4^,S_3/2_, and ^4^F_9/2_. Furthermore, the Er^3+^ ions at the excited state ^4^I_11/2_ could undergo a non-radiative decay to the ^4^I_13/2_ level and subsequently be excited to the ^4^F_9/2_ state by absorbing a second 980 nm photon^24^. Recently, other UCNPs systems, with core-shell nanostructures, were proposed with more complicated upconversion mechanisms^1^ including the non-steady-state upconversion for emissions generated from triply-doped systems and energy transfer mechanism in nanodumbbells nanostructures^25^.

**Figure 5:**
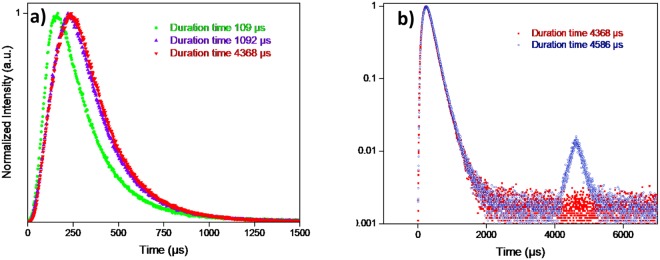
(**a**) Time evolution of upconversion photoluminescence intensity measured under four different excitation duration times (109 *µs* 1092 *µs*, 4368 *µs*, respectively) of the pulsed laser. We observed the dynamic picture of rise time versus excitation duration for 30% Gd^3+^ doped NaYF_4_ sample. We fitted the values of decay lifetimes and rise lifetimes by using algorithm in DAS6 Analysis software, which allows deconvolution analysis of time-domain luminescence data with two exponentials. (**b**) Time evolution of upconversion photoluminescence intensity measured under two different excitation duration times (4368 *µs* and 4586 *µs*, respectively) of the pulsed laser. The temporal evolution of the photoluminescence of NaYF_4_ as a function of time can be described by a Vial’s type equation^32^.

In brief, the unique properties of UCPL are owing to the intra 4f-4f orbital electronic-dipole transitions of lanthanide ions. The long-lived intermediate energy states in lanthanide ions can be attributed to the quantum mechanical forbidden nature of the 4f-4f transition, which allows energy transfers between two or more ions and favour the successive excitations in a single lanthanide ion^26^. Since Yb^3+^ ions have a much broader absorption cross-section than that of Er^3+^ ions for 980 nm light, the ETU process should plays a predominant role among these mechanisms^27^. To investigate the ETU process, Fig. 5(a,b) signify the time evolution of upconversion photoluminescence intensity measured under different excitation duration times of the pulsed laser (109 *µs* 1092 *µs*, 4368 *µs*, 4586 *µs*, respectively). These figures comprise both the rise and decay curves of photoluminescence intensity versus time, which are proportional to the population of the excited states of Er^3+^ as a function of time. In terms of decay lifetime, longer time indicates slower decay rate and favours the accumulation of population^28^.

2 $${\rm{I}}({\rm{t}})={{\rm{I}}}_{1}\exp (\,-\,{\rm{t}}/{{\rm{\tau }}}_{D})+{{\rm{I}}}_{2}\exp (\,-\,{\rm{t}}/{\tau }_{R})$$

where I(t) represent the photoluminescence intensity at a specific time point corresponding to the on and off of the pulsed laser. *τ*_*D*_ is decay lifetime of the UCPL, which can be influenced by the lifetime of excited emitting level and the lifetime of energy levels that feed the emitting level via the ET process. While *τ*_*R*_ represents the rise time, which is also influenced by the lifetime of the emitting state and ET rate. We observed a pronounced rise lifetime dependence on excitation duration for 30% Gd^3+^ doped NaYF_4_ sample. Within the entire measurement range of 11,000 *µs*, as the pulsed laser duration times prolonged from 109 *µs* to 1092 *µs*, the rise time was delayed from 74.6 *µs* to 125.3 *µs*. It was revealed that all GSA/ESA excitation only occurs within the duration of a short laser pulse, when the sample is irradiated^29^. In contrast, the contribution of ETU process can be identified by the slower increase rate and delayed maximum in the time evolution picture of the GSA/ESA and ETU co-existence upconversion photoluminescence. Interestingly, a four times longer duration time, 4368 *µs*, leads to only a slight increased rise time, compared with the 1092 *µs* duration time measurement. With further extension of the duration time to 4586 *µs*, there were negligible changes of rising time. This result could be attributed to the saturation of the intermediate levels^10^. The appearance of a secondary peak at 4600 *µs* in Fig. 5(b) corresponds to the initiation of re-excitation of the ground state when the duration time (4586 *µs*) reached a critical period of time (*T*_*c*_). Thus, in the following measurements, duration time of 4368 *µs* was selected to overcome the restriction of re-excitation effect.

**Figure 6:**
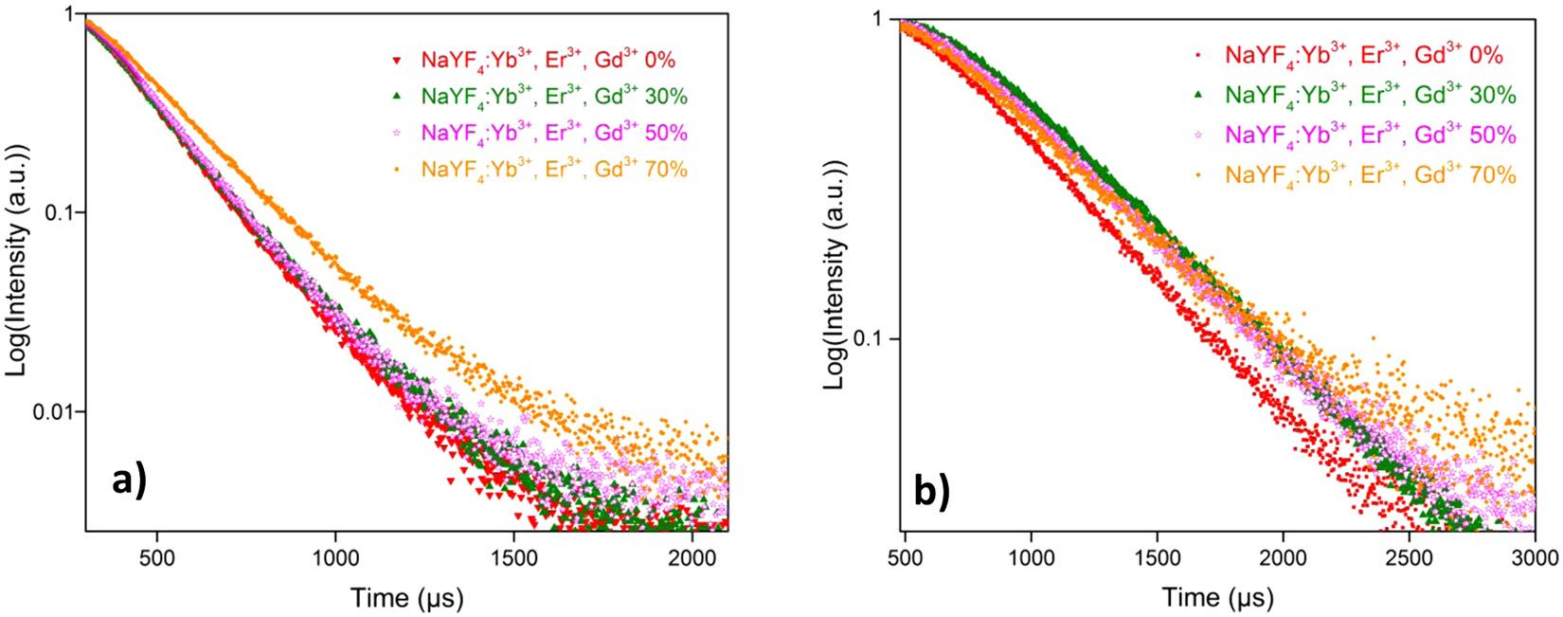
(**a**) Decaycurves for the green emission (540 nm) of NaYF_4_ doped with different molar ratio of Gd^3+^ ions. (**b**) The decay curves for the red emission (656 nm) of NaYF_4_ doped with different molar ratio of Gd^3+^ ions.

In order to demonstrate the Gd^3+^ dopant concentration dependent photoluminescence dynamics, the lifetime decay curves of the green (550 nm) and red (656 nm) emissions of NaYF_4_:Yb^3+^, Er^3+^, Gd^3+^ were measured in samples with increased concentration of Gd^3+^ dopant under excitation of 980 nm pulsed laser, as shown in Fig. 6.

**Figure 7:**
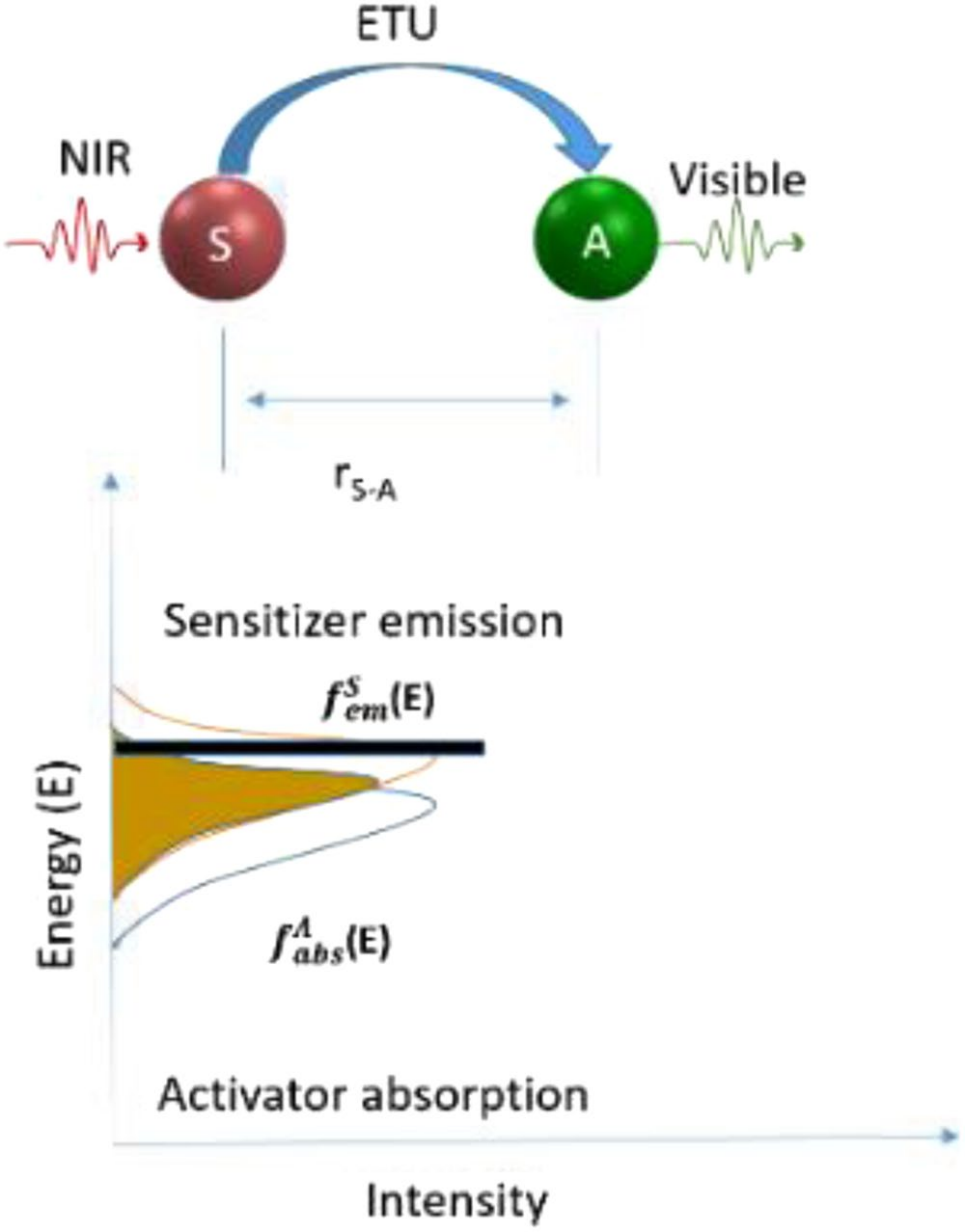
Schematic diagram of energy transfer upconversion ((ETU) in sensitizer to activator, (S-A) coupled system.

The results in Table 1 shows that the decay lifetime *τ* increased with the variation of Gd^3+^ concentration. Clearly, the photoluminescence lifetimes of both green and red emissions were prolonged accordingly as a function of increasing the molar concentration of Gd^3+^ doping in NaYF_4_. In case of samples doped with 30 mol%, 50 mol% Gd^3+^ ions, the green lifetimes increase slightly from 209.62 µs to 211.61 µs, and from 495.1 µs to 499.8 µs for red emission. Theoretically, the lifetime (*τ*) of an excited state is expressed as^30^,

3 $$1/\tau =1/{\tau }_{rad}+1/{\tau }_{nr}+{k}_{ET}$$

where *τ*_*rad*_ represents the radiative decay, *τ*_*nr*_ represents the non-radiative decay lifetime, and *k*_*ET*_ is energy transfer rate. As previously results informed, the upconversion process is dominated by ETU mechanisms in the sensitizer to activator (S-A) couples system (Fig. 7). Dexter’s theory^31^ described the relationship of energy transfer probability (W_*S*-*A*_) and the S-A separation (*r*_S-A_), which can be simplified as,

4 $$\,{{\rm{W}}}_{S \mbox{-} A}\,{\rm{\alpha }}\,\frac{1}{({{R}}_{{S} \mbox{-} {A}})}$$

**Table 1:** Photoluminescence lifetimes of green emission (540 nm) and red emission (656 nm) for NaYF_4_ samples, with different Gd^3+^ molar concentration.

Samples	0% Gd^3+^	30% Gd^3+^	50% Gd^3+^	70% Gd^3+^
Lifetime 540 nm	194.44 µs ± 0.38 µs	209.62 µs ± 0.62 µs	211.61 µs ± 0.82 µs	232.43 µs ± 0.43 µs
Lifetime 656 nm	442.69 µs ± 3.14 µs	495.13 µs ± 2.62 µs	499.84 µs ± 3.04 µs	503.69 µs ± 2.43 µs

This relationship indicates that S-A separation is a significant factor influencing the energy transfer probability. The strong dependence of energy transfer rate on the interionic distance, (*r*_*S-A*_) directly leads to the significance of sensitizer and activator ions concentration in the host of UCNPs. With the increased Gd^3+^ ions doped into the NaYF_4_ nanocrystals, more Y^3+^ and Yb^3+^ ions were substituted by Gd^3+^ in the crystal lattice of NaYF_4_. With the average S-A separation (*r*_*S-A*_) increases accordingly, a decreased energy transfer rate between sensitizer and activator could lead to the prolonged lifetime of NaYF_4_ Nanocrystals.

**Figure 8:**
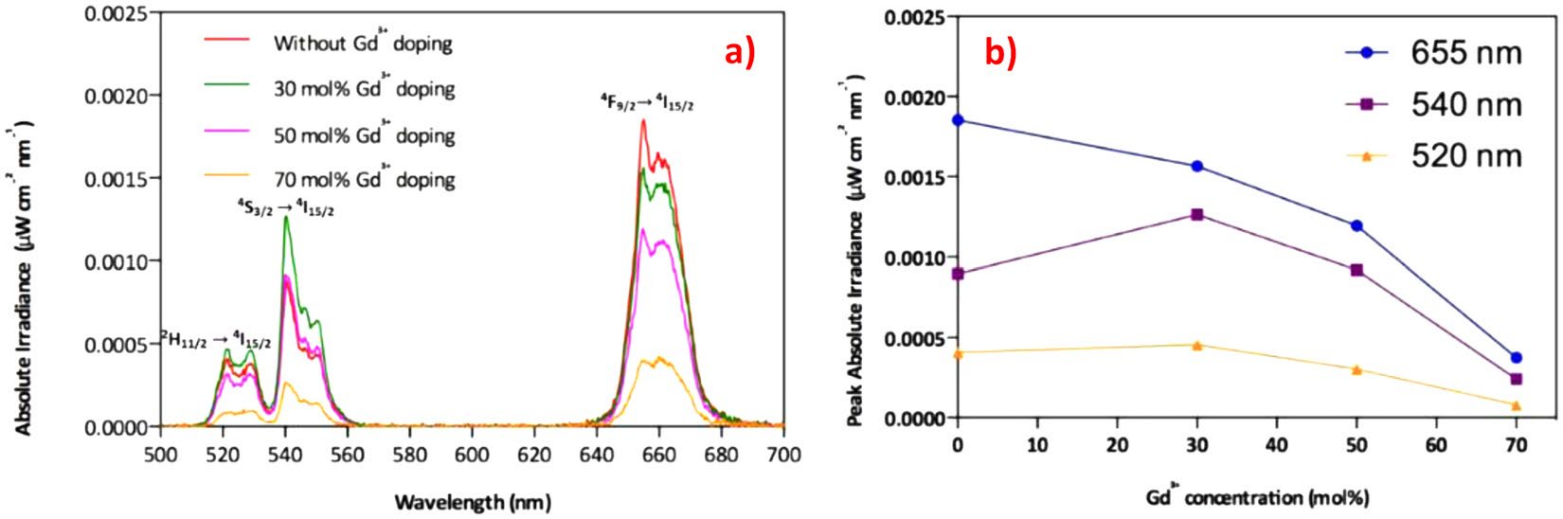
(**a**) demonstrates the absolute irradiance of UC photoluminescence emission spectra of the resulting NaYF_4_:Yb^3+^, Er^3+^ when tuning the Gd^3+^ dopant molar concentration from 0 to 70 mol% upon 980 nm laser excitation. (**b**) shows the UC luminescence emission intensity at 656 nm, 540 nm, and 520 nm versus different Gd^3+^ doping concentration (0, 30 mol%, 50 mol%, and 70 mol%).

To investigate the photoluminescence of NaYF_4_:Yb^3+^, Er^3+^ UCNPs as a function of the Gd^3+^ concentration, the absolute quantum yields of upconversion nanoparticles were measured (Fig. 8a). Clearly, the absolute quantum yield of both green light emission (500 nm–570 nm) and red light emission (620 nm–700 nm) varied as a function of the Gd^3+^ ions doping concentration. The calculated quantum yields at different emission bands with various Gd^3+^ concentrations are summarized in Table S3. In Fig. 8a, the shapes of the emission spectra for the four samples are similar, which suggests the same combination of upconversion pathways for these as-prepared samples. Besides, the intensity ratio of green and red emission demonstrated a remarkable change as a result of the incorporation of Gd^3+^ ions into NaYF_4_:Yb^3+^, Er^3+^ host lattice. With 30 mol% Gd^3+^ doping, the UC luminescence efficiency is enhanced at 540 nm and 520 nm, compared to those without doping. However, the highest emission peak occurs at 656 nm when Gd^3+^ ions are not doped into the NaYF_4_:Yb^3+^, Er^3+^ UC systems. These results reveal that the suitable lanthanide doping and controlled dopant ion concentration are able to modify the UCPL efficiency at selective wavelengths. In the perspective of the ladder-like energy levels in the sensitizer and activator, Gd^3+^ doping induces large local distortion in crystal lattice, and reducing the site symmetry of the activators. Therefore, the probabilities of different pathways in the ETU process could be changed due to the modified lattice symmetry, unit cell parameters and intra-4f transition probability. In addition, the iUCQY were proved to be in correlation with the evolution trend of UCPL lifetimes. Therefore, both the UCPL lifetimes and iUCQY were proved to be influenced by the tailoring of ETU efficiency.

## Conclusions

In summary, we demonstrate that UCPL lifetimes and iUCQY at selective emissions can be tuned by varying the molar concentration of the Gd^3+^ ions. In addition, the properties of UCPL are identified highly sensitive to the energy transfer rate between the sensitizer and activator. Based on the precise control of UCPL lifetimes in the wide range timescale, this doping strategy make it possible to create extra temporal-domain coding dimension, promising significant potential for practical multiplexed applications.

## Electronic supplementary material

Supplementary Information
