# Supplementary material for: Tuning the upconversion photoluminescence lifetimes of NaYF4:Yb3+, Er3+ through lanthanide Gd3+ doping
Source: Sci Rep. 2018 Aug 23;8:12683. doi: 10.1038/s41598-018-30983-9 (PMC6107552; doi:10.1038/s41598-018-30983-9)
Supplement: Supplementary file 1 — Supplementary Information [file 41598_2018_30983_MOESM1_ESM.docx]

**Tuning the upconversion photoluminescence lifetimes of NaYF**4**:Yb**3+**, Er**3+ **through lanthanide Gd**3+ **doping**

Heng Qin1 , Danyang Wu1 , Juna Sathian1 , Xiangyu Xie1 , Mary Ryan1 & Fang Xie1,*

*1Department of Materials and London Centre for Nanotechnology, Imperial College London, Exhibition Road, London, SW7 2AZ, United Kingdoms*

# Emails: [f.xie@imperial.ac.uk](mailto:f.xie@imperial.ac.uk)

# Supplementary

# 2.1 Chemicals

All reagents were of analytical grade. Yttrium(III) nitrate hexahydrate (Y(NO3)3 ∙ 6H2O, Sigma-Aldrich, ≥99.9%), ytterbium(III) nitrate pentahydrate (Yb(NO3)3 ∙ 5H2O, Sigma-Aldrich, ≥99.9%), erbium(III) nitrate pentahydrate (Er(NO3)3 ∙ 5H2O, Sigma-Aldrich, ≥99.9%), europium(III) chloride hexahydrate (EuCl3 ∙ 6H2O, Sigma-Aldrich, ≥99.9%), gadolinium(III) chloride hexahydrate (GdCl3 ∙ 6H2O, Sigma-Aldrich, ≥99.9%), gadolinium(III) oxide (Gd_2_O_3_, Sigma-Aldrich, ≥99.99%), sodium fluoride (NaF, Alfa Aesar, ≥99.99%), oleic acid (OA, Fisher Scientific, ≥97%), ethanol absolute (VWR International, ≥99.7%), and sodium hydroxide (NaOH, VWR International, 40% W/V) were used as supplied.

**2.2 Synthesis of NaYF_4_:Yb^3+^, Er^3+^ co-doped Gd^3+^ Upconversion Nanoparticles**

In a typical synthesis process, the rare earth (RE) nitrate RE(NO_3_)_3_ (Re = Y^3+^, Yb^3+^, and Er^3+^) and gadolinium chloride GdCl_3_ were dissolved in DI water to obtain the aqueous solutions, respectively. Then, a 29.28 mL solution containing 1.952 mmol RE nitrate was mixed with 20 mL ethanol and 25 mL oleic acid under vigorous stirring. The ytterbium nitrate and erbium nitrate solutions were added to the resulting solutions according the calculated quantities. The molar ratio of Yb^3+^:Er^3+^ was controlled to be 20 mol% and 2 mol%, respectively. And the molar ratio of the Gd^3+^ ion dopant was controlled to be 30 mol%, 50 mol% and 70 mol% respectively. The gadolinium chloride aqueous solution was added to the resulting mixture according the calculated quantities. Then, 1 mL NaOH solution and 0.85 g NaF powder were slowly added into the aqueous solution under vigorous stirring. After another vigorous agitation for 30 minutes, the mixture was transferred into a 100 mL Teflon-lined autoclave, and then heated at 200 $℃$ for 20 hours. The resulting white precipitates were obtained by centrifugation, washed with DI water 5 times to remove the residues. The final powder samples were collected and dried at 80 ℃ for 24 hours in a vacuum furnace

**2.3 X-Ray Diffractometer characterisation**

The crystal phase of the products were identified by a X-Ray Diffractometer (XRD) with 2θ range from 10° to 70° at a scanning rate of 4° per minute, with Cu Ka irradiation (k=1.5406 Å). The size A 980 nm CW laser (Beijing Hi-Tech Optoelectronic Co., Ltd.) was used as the excitation source with the power being set at 20 W.cm-2. The upconverting fluorescence spectra were recorded on DCS200PC Photon Counting (Beijing Zolix Instruments Co., Ltd) with single-photon sensitivity through an Omni-λ500 monochromator (Beijing Zolix Instruments Co., Ltd).


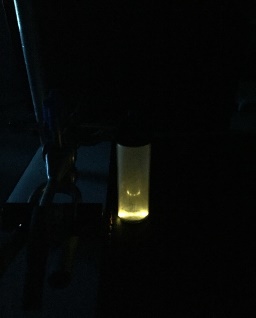


**Figure S1** demonstrates the white precipitates of UC nanoparticles under 980 nm laser excitation at an input intensity of 4.13 W cm^-2^. a yellowish green was obtained under 980 nm laser excitation.

**Table S1*.* shows the reaction parameters and conditions for each sample.**

| **Samples** | **Host/Sensitiser, Activator** | **Gd^3+^ Conc%** | **Molar ratio (Sensitiser: Activator)** | **Reaction parameters** | **Temp. and time** |
| --- | --- | --- | --- | --- | --- |
| 1 | NaYF_4_/Yb^3+^,Er^3+^ | 0 mol % Gd^3+^ | 20:2 | Ethanol 20mL  OA 25 mL  NaOH 1 mL  NaF 0.85 g | 200 $℃$  20 hours |
| 2 | NaYF_4_/Yb^3+^,Er^3+^ | 30 mol % Gd^3+^ |  |  |  |
| 3 | NaYF_4_/Yb^3+^,Er^3+^ | 50 mol% Gd^3+^ |  |  |  |
| 4 | NaYF_4_/Yb^3+^,Er^3+^ | 70 mol% Gd^3+^ |  |  |  |

**2.3 Characterization**

Table S2*.* illustrates the summary of the phase, size, and size distribution of the NaYF_4_:Yb^3+^, Er^3+^ (20, 2) nanocrystals with various concentrations of Gd^3+^ dopant ions (0 mol%, 30 mol%, 50 mol%, and 70 mol%).

| **Sample** | **Coc. of Gd^3+^ doping** | **Phase** | **Width (nm)** | **Length (nm)** |
| --- | --- | --- | --- | --- |
| **NaYF_4_:Yb^3+^, Er^3+^** | 0 mol% | Cubic | 50-100 | 50-100 |
|  |  | Hexagonal | 300-500 | 1900-2200 |
| **NaYF_4_:Yb^3+^, Er^3+^** | 30 mol% | Hexagonal | 200-450 | 700-1000 |
| **NaYF_4_:Yb^3+^, Er^3+^** | 50 mol% | Hexagonal | 130-490 | 600-950 |
| **NaYF_4_:Yb^3+^, Er^3+^** | 70 mol% | Hexagonal | 150-550 | 200-700 |

**Table S3 Absolute Internal Upconversion Quantum Yield values at different wavelengths (521 nm, 541 nm, 656 nm and total) of UCNPs doped with various Gd^3+^ concentrations.**

| **Gd^3+^ Conc.%** | **0%** | **30%** | **50%** | **70%** |
| --- | --- | --- | --- | --- |
| **521 nm** | 0.196% | 0.177% | 0.116% | 0.037% |
| **541nm** | 0.338% | 0.379% | 0.266% | 0.081% |
| **656 nm** | 1.445% | 0.991% | 0.720% | 0.276% |
| **Totol** | 1.980% | 1.548% | 1.102% | 0.394% |
